# Mechanistic Approach for Thioridazine-Induced Hepatotoxicity and Potential Benefits of Melatonin and/or Coenzyme Q10 on Freshly Isolated Rat Hepatocytes

**Published:** 2018

**Authors:** Aziz Eftekhari, Elham Ahmadian, Yadollah Azarmi, Alireza Parvizpur, Javad Khalili Fard, Mohammad Ali Eghbal

**Affiliations:** a *Department of Pharmacology and Toxicology, Maragheh University of Medical Sciences, Maragheh, Iran.*; b *Toxicology Research Center, Maragheh University of Medical Sciences, Maragheh, Iran.*; c *Drug Applied Research Center, Tabriz University of Medical Sciences, Tabriz, Iran. *; d *Department of * *Pharmacology and Toxicology, School of Pharmacy, Tabriz University of Medical Sciences, Tabriz, Iran. *; e *Students’ Research Committee, Tabriz University of Medical Sciences, Tabriz, Iran.*

**Keywords:** Thioridazine, Hepatotoxicity, ROS formation, Oxidative stress, Mitochondrial/lysosomal dysfunction

## Abstract

Thioridazine (TZ) is used mainly in the treatment of schizophrenia. However, hepatotoxicity as a life-threatening adverse effect is associated with its clinical use. In this context, we examined the cytotoxic mechanisms of TZ on freshly isolated rat hepatocytes to better understanding of the pathogenesis of TZ-induced hepatotoxicity. Hepatocytes were prepared by the method of collagenase enzyme perfusion via the portal vein. The level of parameters such as cell death, reactive oxygen species (ROS) formation, lipid peroxidation (LPO), mitochondrial membrane potential (MMP), lysosomal membrane integrity and cellular glutathione (GSH) content in TZ-treated and non-treated hepatocytes were determined and the mentioned markers were assessed in the presence of Coenzyme Q10 and/or melatonin. Results showed that TZ caused an increase in ROS formation as well as induction of LPO and GSH depletion. Moreover, mitochondria and lysosomes seem to be targets of TZ-induced toxicity. The administration of Coenzyme Q10 and/or melatonin efficiently decreased the rate of ROS formation, LPO and improved cell viability, MMP, GSH level and lysosome membrane integrity. This study proposes the possible protective role of Coenzyme Q10 and/or melatonin against TZ-induced cellular injury probably through their radical scavenging properties and their effects on mitochondria and lysosomes.

## Introduction

Thioridazine (TZ), a phenothiazine derivative, as a potent antipsychotic drug is still used in the treatment of psychotic disorders and also has antimicrobial and anti-cancer properties (1, 2). Despite its therapeutic benefits, many authors have reported side effects including cardiotoxicity, retinopathy and hepatotoxicity (2-4).

Although the precise mechanism of TZ-induced liver damage is not clear yet, this adverse effect has been attributed to the induction of oxidative stress and reactive metabolites of the drug (3, 5). To avoid TZ-induced toxicity, no specific protecting agent has been described. 

Oxidative stress is tightly connected to the pathogenesis of various disease and chemical induced toxicities (6-9). Multiple studies and our previous laboratory reports provided strong evidence that antioxidants including melatonin are important hepatoprotective agents with a broad spectrum in drug-induced liver toxicities (8-11). Melatonin (N-acetyl-5-metyoxytryptamine), as a potent endogenous antioxidant, has numerous physiological and biochemical functions (12). The protective effects of melatonin could be due to its up regulatory effects on the antioxidant enzymes that control reactive oxygen species (ROS) production and reduce lipid peroxidation (LPO) (12). Also, melatonin induces the activity of the glutamylcysteine synthetase, which stimulates the production of the other intracellular antioxidant, glutathione (13). 

Coenzyme Q10 (CoQ10) or ubiquinone, is a well-known vitamin-like substance that acts as a proton–electron carrier in the mitochondrial electron transport chain which consequently supplies the energy need of the cell (ATP ) (14). It has been shown that CoQ10 can interact directly with ROS and LPO. Also, CoQ10 is the precursor of different endogenous antioxidants (cellular vitamin E and C regeneration and increment of glutathione reductase and superoxide dismutase levels) (15).

Literature search has fetched several reports demonstrating the protective effects of CoQ10 on drug/xenobiotic-induced hepatotoxicity (16, 17). Beneficial impacts of this agent have been shown in conditions characterized by decreased cellular GSH content, oxidative stress and mitochondrial membrane collapse (16, 17).

The major objective of this study was not only to investigate the exact underlying mechanism of TZ hepatotoxicity, but also to evaluate the potential effects of CoQ10 and/or melatonin against TZ-induced cellular damage in isolated rat hepatocytes. Therefore, we aimed to evaluate the influence of TZ on isolated rat hepatocytes by assessing alterations in cell viability, ROS formation, LPO, GSH level, mitochondrial/lysosomal function and the potential effects of CoQ10 and/or melatonin on the development of TZ-induced hepatotoxicity and oxidative stress in isolated rat liver cells. 

## Experimental

*Chemicals*

Melatonin, 4-2-hydroxyethyl-1-piperazineethanesulfonic acid (HEPES) and oxidized glutathione (GSSG) were obtained from Acros (New Jersey, USA). Thiobarbituric acid (TBA) was purchased from Serva (Heidelberg, Germany). Cimetidine was obtained from Medisca Pharmaceutique Inc. (Montreal, Canada). Bovine serum albumin was purchased from the Roche diagnostic corporation (Indianapolis, IN, USA). Trypan blue, GSH and Phenobarbital were purchased from Merck Chemical Company (Darmstadt, Germany). Type II collagenase and all other chemicals were obtained from Sigma–Aldrich chemical Company (St. Louis, MO, USA).

*Experimental animals*

Adult male Sprague-Dawley rats (250–300 g) were kept in standard ventilated plastic cages with 12 h light cycle and ambient temperature of 20-22 °C with a 50-60% relative humidity. Animals were fed a normal standard chow diet and water *ad libitum*. The animals received humane care according to the ethical guidelines of National Institute of Health (NIH publication No. 85-23, revised 1985). All experiments were conducted according handling protocol of Tabriz University of Medical Sciences, approved by a Committee of Animal Experimentation.

*Isolation of Rat Hepatocytes*

Collagenase perfusion method was used to isolate rat hepatocytes as described previously (18)*.* Isolated hepatocytes (10 mL, 10^6^ cells/mL) were incubated in the Krebs-Henseleit buffer (pH = 7.4) in continuously rotating round bottom flasks supplemented with 12.5mM HEPES, under an atmosphere of carbogen gas ( 95% O_2_ and 5% CO_2_) in a 37 °C water bath (18). Hepatocytes were preincubated for 30 min prior to addition of chemicals. We used EC_50_ concentrations for TZ (50 µM) in order to prevent either nontoxic or very toxic conditions in this study. According to the ACMS (Accelerated cytotoxicity screening technique) method the EC_50_ of a chemical in hepatocyte is defined as the concentration which decreases the hepatocyte viability down to 50% following 120 min incubation (4). 

The doses of CoQ10 was chosen from similar studies that were conducted on freshly isolated rat hepatocytes (17). An optimum effective dose of melatonin that provided suitable protection was found to be 400 µM. To prepare CYP450-inhibited hepatocytes, cimetidine was added 30 min before other reagents to the flasks containing hepatocytes (19, 20). Glutathione (GSH)-depleted hepatocytes were prepared by preincubation of hepatocytes with 200 µM 1-bromoheptane for 30 min as described before (21). 

*Cell Viability*

After the isolation process, hepatocytes viability was determined microscopically by the trypan blue (0.1% w/v) exclusion test (18). Cell viability was assessed every 60 min during 180 min of incubation period. Approximately 85 % to 90 % of hepatocytes were viable before use.

*Determination of ROS*

The rate of TZ-induced ROS formation was determined by adding of 2.7-dichlorofluorescein-diacetate (DCFH-DA) to the incubation medium (22). DCFH-DA penetrates hepatocytes and becomes hydrolyzed by an intracellular esterase to form dichlorofluorescein (DCFH) which reacts with intracellular ROS to form the highly fluorescent 2, 7-dichlorofluorescein that effluxes the cell. Briefly, DCFH-DA (1.6 μM) was added to the hepatocyte incubation and aliquots (1 mL) were withdrawn at 15, 30 and 60 min time points after TZ treatment. These samples were then centrifuged for 1 min at 3000*g*. The fluorescence intensity of DCFH was measured using a Jasco R_ FP-750 spectrofluorometer (Jasco Corporation, Tokyo, Japan) with excitation and emission wavelengths of 500 and 520 nm, respectively (22).

*Determination of Lipid peroxidation*

LPO which has an undesirable effect on cell membrane or subcellular organelles membranes is often a subsequent event of drug/xenobiotic-induced oxidative (23). Hepatocytes LPO was determined by measuring the thiobarbituric acid reactive substances (TBARS) that were formed during the decomposition of lipid hydroperoxide. Briefly, 250 μL trichloroacetic acid (TCA 70% w/v) was added to 1 ml of hepatocytes suspension (10^6^ cells) and centrifuged for 15 min at 3000*g*; then 1ml of thiobarbituric acid (TBA 0.8% w/v) was added to supernatant and boiled for 20 min. The absorbance was determined at 532 nm in an Ultrospec® 2000 UV spectrophotometer (10).

*Determination of mitochondrial membrane potential*

Rhodamine 123, the cationic fluorescent dye, accumulates in intact mitochondria by facilitated diffusion and is used as a probe for mitochondrial membrane potential (MMP) loss. When MMP is altered by any xenobiotic, the diffusion of the dye ends up leading to accretion of the amount of rhodamine 123 in media. For this purpose 1mL samples of the cell suspension were picked up and centrifuged at 1000 *g *for 1 min at the given times. Afterwards, the cell pellet was resuspended in 2 mL of fresh incubation medium containing 1.5 μM of rhodamine 123 and incubated at 37 °C in a water bath while a gentle shake was done. Hepatocytes separation was done by centrifugation at 3000 *g *for 1 min and the amount of rhodamine 123 left in the incubation medium was determined using a Jasco FP-750 fluorescence spectrophotometer set at 490 nm excitation and 520 nm emission wavelengths (9).

*Measurement of intracellular GSH and GSSG*

Derivatization with iodoacetic acid and 1-fluoro-2,4-dinitrobenzene, by HPLC (24) using a µBondapak NH2 column (Water Associates, Milford, MA) was utilized to measure reduced glutathione (GSH) and oxidized glutathione (GSSG) contents in isolated hepatocytes. The procedure is based upon the principal formation of *S*-carboxymethyl derivatives of free thiols with iodoacetic acid followed by conversion of free amino groups to 2, 4-dinitrophenyl derivatives by reaction with 1-fluoro-2, 4-dinitrobenzene (FDNB). Nano-molar detection of GSH and GSSG levels is feasible with this method. In brief, 0.8 mL of the cell suspension was spun at 50 g for 40 s, and the cell pellet was resuspended in 0.8 mL of fresh Krebs-Hensleit medium. A volume of 0.2 mL of 25% metaphosphoric acid was added to deproteinize the sample followed by centrifugation at 100×*g *for 5 min. Then, 0.5 mL of supernatant and 0.05 mL of iodoacetic acid were mixed in the presence of excess sodium bicarbonate and left in the dark at room temperature for 1 h. In the next step, 0.5 mL of FDNB solution (1.5%, v/v in ethanol) was added to the sample, left in the dark for 24 h at room temperature and then was analyzed by HPLC (18).

*Lysosomal membrane integrity assay*

Hepatocyte lysosomal membrane stability was measured through redistribution of the fluorescent dye, acridine orange (25). Acridine orange (5 µM) pre-stained cell suspension (0.5 mL) were separated from the incubation medium by 1 min centrifugation at 800 g with a further resuspension in 2 mL of fresh incubation buffer. In order to eliminate the fluorescent dye, washing process was repeated twice. Acridine orange redistribution in the cell suspension was finally determined fluorimetrically using a Shimadzu RF5000U fluorescence spectrophotometer set at 495 nm excitation and 530 nm emission wavelengths.

*Statistical analysis*

The results are shown as the Mean ± SEM for at 3 three independent experiments. Statistical analysis for the control and experimental groups was performed by a one-way analysis of variance (ANOVA) followed by Tukey’s* post hoc *test to assess significance. Results with values for *p *< 0.05 were considered statistically significant.

## Results

*Cytotoxicity Caused by TZ*

**Figure 1:**
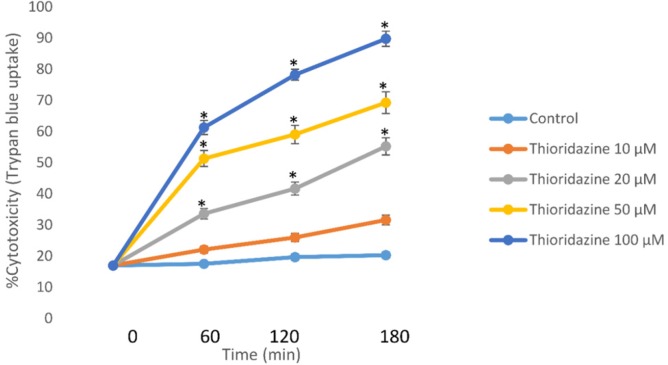
Concentration-response curves of thioridazine-induced cytotoxicity toward isolated rat hepatocytes. Isolated rat hepatocytes (10^6^ cells/ mL) were incubated at 37 °C in rotating round bottom flasks with 95% O_2_ and 5% CO_2_ Henseleit buffer (pH 7.4). Data are given as Mean ± SEM for three independent experiments. *Different from control group (*p* < 0.05).

Incubation of isolated rat hepatocytes for 120 min at 37 °C with 50 μM TZ caused an approximate 50% cell death in a concentration-dependent manner as measured by the trypan blue exclusion assay through dose-response curve (Figure 1).

This EC50 value (50 μM) of TZ was used to investigate potential cytotoxic mechanisms and alterations in biochemical markers. As presented in Table 1, 50 µM of TZ significantly increased hepatocytes membrane lysis compared to control hepatocytes. Melatonin and/or CoQ10 were added to hepatocytes to determine their effects on TZ-induced toxicity. The optimum doses for melatonin and/or CoQ10 were evaluated to be 400 µM and 200 µM respectively which effectively reduced TZ-induced cell death (Table1). Furthermore, preincubation of hepatocytes with cimetidine (2 mM) as a CYP450 inhibitor caused a significant decline in the percentage of cell death (Table1). As shown in table 1, TZ was more toxic towards glutathione depleted hepatocytes (with 1-bromoheptane) compared to the normal control cells (*p* < 0.05) (Table 1).

**Table1:** Effect of antioxidants, cimetidine and GSH depletion on thioridazine-induced hepatocyte toxicity

***Treatment***	** Cytotoxicity %**		
	**Incubation time**		
	**60 min**	**120 min**	**180 min**
Control (only hepatocytes)	18 ± 3	21 ± 2	24 ± 2
Thioridazine (50µM)	49 ± 4^a^	58 ± 4^a^	63 ± 4^a^
+Melatonin (400 µM)	28 ± 6^b^	31 ± 3^b^	33 ± 5^b^
+ CoQ10 (200 µM)	25 ± 5^b^	28 ± 4^b^	30 ± 4^b^
+Cimetidine (2mM)	23 ± 5^b^	26 ± 3^b^	28 ± 3^b^
1-bromoheptane control	20 ± 3	23 ± 3	25 ± 4
+ Thioridazine (50µM)	55 ± 3	75 ± 3^b^	84 ± 4 b

Hepatocytes (10^6^ cells/mL) were incubated in Krebs–Henseleit buffer, pH 7.4, at 37 °C For 180 min following the addition of thioridazine (50 µM). Cell viability was assessed by trypan blue exclusion test (18). GSH depleted hepatocytes (with 1-bromoheptane) were prepared as described by Khan *et al. *(21). Results are demonstrated as mean ±SEM. of at least three different experiments.

a Significant difference as compared to the control hepatocytes (*p *< 0.05).

b Significant difference as compared to the Thioridazine-treated hepatocytes (*p*< 0.05).

*TZ-induced ROS formation*

Treatment of hepatocytes with 50 μM TZ led to a significant amount of ROS formation while administration of 400 μM melatonin, 200 μM CoQ10 and CYP inhibitor (cimetidine 2mM) effectively reduced ROS generation (Table 2). Also, as shown in Table 3, TZ-induced ROS formation significantly was increased in glutathione-depleted hepatocytes.

**Table 2:** Effect of antioxidants, cimetidine and GSH depletion on thioridazine-induced ROS formation

***Treatment***	**ROS formation (DCF: fluorescent intensity units)**		
	**Incubation time**		
	**15 min**	**30 min**	**60 min**
Control (only hepatocytes)	95 ± 2	107± 3	120 ± 5
Thioridazine (50µM)	119 ± 3^a^	148 ± 3^a^	174 ± 2^a^
+Melatonin (400 µM)	103 ± 2^b^	109 ± 4^b^	134 ± 3^b^
+ CoQ10 (200 µM)	101 ± 3^b^	112 ± 3^b^	131 ± 5^b^
+Cimetidine (2mM)	106 ± 4^b^	110 ± 5^b^	128 ± 3^b^
1-bromoheptane control	98 ± 3	112 ± 2	125 ± 3
+Thioridazine (50µM)	173 ± 3^b^	236± 4 b	342 ± 2 b

Hepatocytes (10^6^ cells/mL) were incubated in Krebs–Henseleit buffer pH7.4 at 37 °C for 60 min following the addition of thioridazine (50 µM). ROS generation measured by dichlorofluorescein (DCF) formation method expressed as fluorescent intensity units (22). GSH depleted hepatocytes (with 1-bromoheptane) were prepared as described by Khan *et al.* (21). Values are expressed as mean ± S E M of three separate experiments.

a Significant difference in comparison with control hepatocytes (*p *< 0.05).

b Significant difference in comparison with thioridazine-treated hepatocytes (*p *< 0.05).

*The Effects of TZ on Lipid Peroxidation*

**Figure 2:**
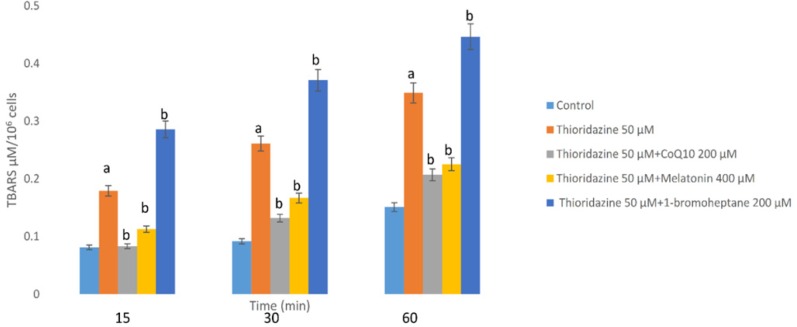
Hepatocytes (10^6^ cells/mL) were incubated in Krebs–Henseleit buffer pH 7.4, at37 °^C^ for 60 min following the addition of thioridazine (50 µM). TBARS formation was expressed as µM concentrations (10). GSH depleted hepatocytes (with 1-bromoheptane) were prepared as described by Khan *et al*. (21). Values are expressed as mean ± SEM of three separate experiments

^a^Significant difference in comparison with control hepatocytes (*p *< 0.05).

^b^Significant difference in comparison with thioridazine -treated hepatocytes (*p *< 0.05).

As a consequence of ROS generation and oxidative stress, peroxidation of membrane lipids was increased by TZ which was concurrent with the production of TBARS. As shown in Figure 2, TZ-induced toxicity was associated with the peroxidation of lipids. Again treatment with antioxidants (melatonin and/or CoQ10) decreased the production of TBARS significantly (*p < *0.05). In the hepatocytes whose glutathione had been depleted by 1-bromoheptane, TZ- associated LPO was increased dramatically (Figure 2).

*The Effects of TZ on Mitochondrial Membrane Potential*

**Figure 3:**
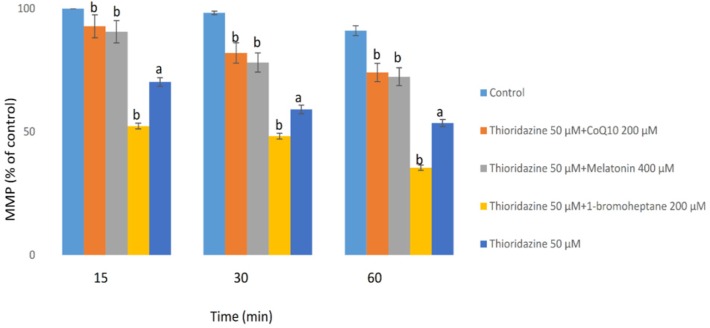
Hepatocytes (10^6^ cells/mL) were incubated in Krebs–Henseleit buffer, pH 7.4, at 37 °C for 60 min following the addition of thioridazine (50µM). Mitochondrial membrane potential was determined as the percentage of mitochondrial rhodamin reuptake between control and treated cells (9). GSH depleted hepatocytes (with 1-bromoheptane) were prepared as described by Khan *et al.* (21). Values are expressed as mean ± SEM of three separate experiments

^a^ Significant difference in comparison with control hepatocytes (*p* < 0.05).

^b^ Significant difference in comparison with thioridazine-treated hepatocytes (*p* < 0.05).

The MMP is the major driving force for ATP production and alterations on this parameter were measured with a potentiometric fluorescent probe rhodamine 123, which can accumulate in negatively charged mitochondrial matrix. As shown in figure 3, TZ (50 μM) dramatically reduced the mitochondrial membrane potential (MMP) which is a bold marker of mitochondrial toxicity. TZ-induced mitochondrial membrane collapse was effectively prevented by CoQ10 and/or melatonin (*p < *0.05) (Figure 3). Depletion of glutathione reservoirs markedly increased TZ-induced mitochondrial depolarization (Figure 3).

*The Effects of TZ on GSH/GSSG Levels*

As shown in table 3, after incubation with TZ (50 μM), the intracellular GSH level was decreased and the depletion of GSH was subsequently followed by an increase in glutathione disulfide (GSSG) level. In contrast, treatment of hepatocytes with CoQ10 and/or melatonin significantly (*p *< 0.05) prevented not only GSH depletion, but also the increment of GSSG levels caused by TZ. Both of the antioxidants (CoQ10 and melatonin) did not cause significant changes (*p* < 0.05) in hepatocytes GSH/GSSG status at the concentrations used when administered alone (data not shown).

**Table 3:** Effect of antioxidants on Intracellular GSH and GSSG during thioridazine-induced hepatocyte injury

**Addition**	**Intracellular GSH (nmol/10** ^6^ **cell) Intracellular GSSG (nmol/10** ^6^ **cell)**						
**Incubation Time (min) **	**15**	**30**	**60**	**15**		**30**	**60**
Control	56.39± 4	52.26± 5	48.31 ± 3	5.74 ± 4	6.98 ± 4		10.15 ± 4
Thioridazine50µM	48.13± 5^a^	45.29 ± 3^a^	39.34 ± 6^a^	10.13 ±7*	14.64 ± 8*		19.47 ± 4*
+Melatonin 400 µM	55.75± 3^b^	51.47± 5^b^	48.85 ± 6^b^	6.92 ± 3^b^	8.57 ± 5^b^		11.61 ± 4^b^
+ CoQ10 200 µM	56.14± 6^b^	51.58± 4^b^	47.64 ± 5^b^	6.07 ± 3 b	7.13 ± 5 b		10.41 ± 6 b

Hepatocytes (10^6^ cells/mL) were incubated in Krebs–Henseleit buffer pH 7.4 at 37 °C for 1 h following the addition of thioridazine (50 µM). Intracellular GSH and GSSG was determined by HPLC analysis (18).

a Different from control group (*p *< 0.05).

b Significantly different from thioridazine-treated hepatocytes (*p *< 0.05).Values shown are the means ± SEM of three separate experiments.

*TZ-induced lysosomal membrane stability change*

**Figure 4:**
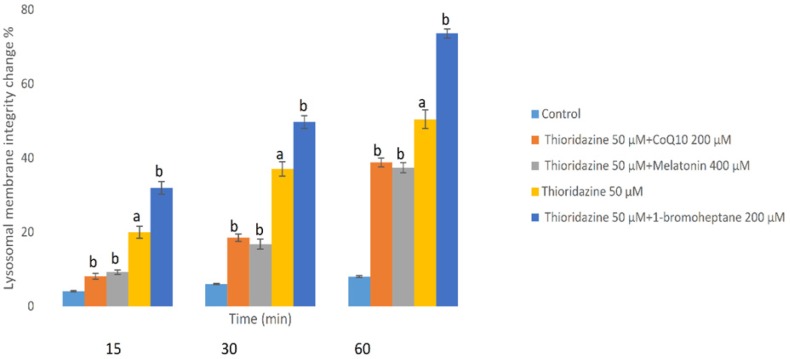
Hepatocytes (10^6^ cells/mL) were incubated in Krebs–Henseleit buffer pH 7.4 at 37 °C for 1 h following the addition of thioridazine (50 µM). Lysosomal membrane fragility was measured as fluorescent intensity unit of diffused cytosolic green fluorescence induced by acridine orange following the redistribution from lysosomes into cytosol in acridine orange loaded hepatocytes(25). GSH depleted hepatocytes (with 1-bromoheptane) were prepared as described by Khan *et al*. (21). Values are expressed as mean ± SEM of three separate experiments (n = 3).

^a^ Significant difference in comparison with control hepatocytes (*p* < 0.05).

^b^ Significant difference in comparison with thioridazine-treated hepatocytes (*p* < 0.05).

Loading of hepatocyte lysosomes with acridine orange following hepatocyte treatment with TZ caused a significant redistribution of acridine orange from lysosomes to cytosol within 60 min, indicating leakiness of lysosomal membrane (Figure 4). TZ-induced acridine orange redistribution was again prevented by CoQ10 and/or melatonin (Figure 4). Also, depletion of hepatocytes glutathione again enhanced the acridine orange release from the lysosomes in TZ-treated hepatocytes. In this experiment, antioxidants (CoQ10 and melatonin) did not exhibit any significant effect *p* > 0.05) on the aforementioned hepatocytes cytotoxicity markers at used concentrations when used alone (data not shown).

## Discussion

TZ, as a piperidine-type phenothiazine antipsychotic is used in the management of psychosis but there are toxicological implications associated with its intake such as hepatotoxicity and cardiotoxicity (2, 26). It was demonstrated that phenothiazines-induced cytotoxicity was associated with oxidative stress, apoptosis and mitochondrial dysfunction (18,27-29). Otręba1 *et al.* demonstrated that TZ stimulates the generation of reactive species and also alterations in the activity of superoxide dismutase and catalase enzymes were also stated by other authors (1, 4). The present work provides the first *in-vitro* study on the mechanisms involved in TZ-induced oxidative stress and organelle disorders using isolated rat hepatocytes. Our results showed that oxidative stress is firmly involved in the pathogenesis of TZ-induced cytotoxicity and it was prevented by cimetidine, CoQ10 and/or melatonin which is in harmony with previous studies (30-33). Also, we confirmed that TZ-induced ROS formation was prevented by CoQ10 and/or melatonin. 

Even though the mechanisms of TZ cytotoxicity is not precisely elucidated so far, there are some evidence that reactive metabolites may play an important role in this toxicity (3). Our results demonstrated that inhibition of CYP with cimetidine decreased TZ-induced cytotoxicity indicating that bioactivation of the drug by CYP450 could surge toxicity in hepatocytes; this is in accordance with previous studies (20).

LPO as a subsequent event of oxidative stress in biological systems increases the membrane permeability and leads to cell death (34). Findings presented by Dhaunsi *et al. *showed that TZ-induced LPO changes the function of certain myelin-related enzymes (35). The present study confirmed that treatment of rat hepatocytes with TZ led to the induction of LPO which was prevented by antioxidants (CoQ10 and/or melatonin), strongly proposing the involvement of oxidative stress in the cytotoxicity of TZ in hepatocytes.

Mitochondria as cell power plants are the main sites of the drug-induced cytotoxicity. ROS are mainly produced in the mitochondria as by-products of respiration carried through the electron transport chain. Numerous studies reported that antipsychotic drugs could inhibit mitochondrial electron transport chain function and activate the mitochondrial permeability transition (MPT) connected with the liberation of cytochrome c and cell death (36, 37). Also, de Faria *et al.* reported that TZ has more potent mitochondrial toxicity among the other phenothiazine derivatives (27). Consistent with these findings, we demonstrated the implication of mitochondrial impairment in the pathogenesis of TZ-induced cytotoxicity (Figure 3).

CoQ10 is a redox-active lipophilic ROS scavenger and found in various cellular organelles such as mitochondria, lysosomes and Golgi vesicles (38). It takes role as a cofactor in the electron transport chain of mitochondria and plasma membranes, transferring free electrons from complexes I and II to complex III during energy-producing metabolic pathways (39). Exogenous CoQ10 supplementation is used to treat a variety of diseases such as diabetes mellitus (40), carcinomas (41) and mitochondrial diseases (42). Moreover, it has been revealed that CoQ10 has hepatoprotective effects against drug/xenobiotic-promoted cellular damage and oxidative stress in several cases such as statins (10), acetaminophen (16), rizatriptan (43), CCl_4_ (44).

Melatonin as a biosynthetic precursor of endogenous antioxidants promotes mitochondrial functions in xenobiotic-induced hepatotoxicity and oxidative stress (45). Significant decrease in MMP collapse with antioxidants (CoQ10 and/or melatonin) in our study, suggested that MMP decrease was a consequence of oxidative stress which are in line with previous investigations that these potent antioxidants, act either as scavengers of free radicals and/or also might be beneficial for improving mitochondrial oxidative phosphorylation (16).

GSH as an intracellular antioxidant, acts through protecting cells from intracellular ROS formation and LPO by keeping protein thiol groups from oxidation via radicals, hence from the current point of view, glutathione depletion is a bold marker of oxidative stress. Our findings indicate that when hepatocytes were treated with TZ, depletion of glutathione was occurred subsequent to ROS formation and LPO. Furthermore, depletion of GSH led to further cytotoxicity in the context of ROS formation and MMP collapse. These outcomes support the assumption that administered CoQ10 and/or melatonin act substantially to inhibit glutathione depletion and combat destructive effects of LPO and ROS formation.

As a consequence of intracellular ROS production in mitochondria, large amounts of intracellular H_2_O_2_ are generated and due to the incomplete detoxifying processes, this lipophilic compound easily diffuses in the cell and passes through lysosomal membrane. Fenton-type reaction between hydrogen peroxide and lysosomal Fe^3+^, forms a highly reactive hydroxyl radical that leads to the LPO of membrane and reduces lysosomal integrity with subsequent release of the its enzymatic content into the cytosol (46). Abovementioned destructive pathway results in amplification of oxidative damage from mitochondria and redox-active iron rich lysosomes. Also, Zong *et al*. reported that phenothiazine-induced mitochondrial depolarization and lysosomal dysfunction is cytotoxic in small cell lung carcinoma (47).

Mitigation of TZ-induced lysosomal damage by melatonin and/or CoQ10 reveal the counter action of aforementioned agents on lysosomal membrane permeability and all subsequent stages of the apoptotic cascade which are in agreement with previous studies. 

## Conclusion

Our results indicate that TZ-induced liver damage causes oxidative-stress and subsequent toxic events including: LPO, mitochondrial membrane potential collapse, GSH depletion and lysosomal membrane injury. Therefore, in addition to CoQ10 and/or melatonin, the use of other antioxidants against TZ-induced hepatotoxicity is the forthcoming research field to be documented.

## References

[1] Otręba M, Beberok A, Wrześniok D, Rok J, Buszman E (2015). Effect of thioridazine on antioxidant status of HEMn-DP melanocytes. Naunyn Schmiedebergs Arch. Pharmacol.

[2] Amaral L, Molnar J (2012). Potential therapy of multidrug-resistant and extremely drug-resistant tuberculosis with thioridazine. In-vivo.

[3] Wen B, Zhou M (2009). Metabolic activation of the phenothiazine antipsychotics chlorpromazine and thioridazine to electrophilic iminoquinone species in human liver microsomes and recombinant P450s. Chem. Biol. Interact.

[4] Rukmini M, D›souza B, D›souza V (2004). Superoxide dismutase and catalase activities and their correlation with malondialdehyde in schizophrenic patients. Indian J. Clin. Biochem.

[5] Tolosa L, Gomez-Lechon MJ, Perez-Cataldo G, Castell JV, Donato MT (2013). HepG2 cells simultaneously expressing five P450 enzymes for the screening of hepatotoxicity: identification of bioactivable drugs and the potential mechanism of toxicity involved. Arch. Toxicol.

[6] Eftekhari A, Ahmadian E, Panahi-Azar V, Hosseini H, Tabibiazar M, Maleki Dizaj S (2018). Hepatoprotective and free radical scavenging actions of quercetin nanoparticles on aﬂatoxin B1-induced liver damage: in-vitro/in-vivo studies. Artif. Cells Nanomed. Biotechnol.

[7] Ahmadian E, Babaei H, Nayebi AM, Eftekhari A, Eghbal MA (2017). Mechanistic approach for toxic effects of bupropion in primary rat hepatocytes. Drug Res.

[8] Eftekhari A, Ahmadian E, Azarmi Y, Parvizpur A, Hamishehkar H, Eghbal MA (2016). In-vitro/vivo studies towards mechanisms of risperidone-induced oxidative stress and the protective role of coenzyme q10 and n-acetylcysteine. Toxicol. Mech. Methods.

[9] Ahmadian E, Eftekhari A, Khalili Fard J, Babaei H, Mohajjel Nayebi A, Mohammadnejad D, Eghbal MA (2017). In-vitro and in-vivo evaluation of the mechanisms of citalopram-induced hepatotoxicity. Arch. Pharm. Res.

[10] Ahmadian E, Babaei H, Mohajjel Nayebi A, Eftekhari A, Eghbal MA (2016). Venlafaxine-Induced Cytotoxicity Towards Isolated Rat Hepatocytes Involves Oxidative Stress and Mitochondrial/Lysosomal Dysfunction. Adv. Pharm. Bull.

[11] Mandegary A, Saeedi A, Eftekhari A, Montazeri V, Sharif E (2013). Hepatoprotective effect of silyamarin in individuals chronically exposed to hydrogen sulfide; modulating influence of TNF-α cytokine genetic polymorphism. DARU J. Pharm. Sci.

[12] Reiter RJ, Tan DX, Maldonado MD (2005). Melatonin as an antioxidant: physiology versus pharmacology. J. Pineal. Res.

[13] Winiarska K, Fraczyk T, Malinska D, Drozak J, Bryla J (2006). Melatonin attenuates diabetes-induced oxidative stress in rabbits. J. Pineal. Res.

[14] Littarru GP, Tiano L (2010). Clinical aspects of coenzyme Q10: an update. Nutrition.

[15] Carocho M, Ferreira IC (2013). A review on antioxidants, prooxidants and related controversy: natural and synthetic compounds, screening and analysis methodologies and future perspectives. Food Chem. Toxicol.

[16] Eftekhari E, Ahmadian E, Azami A, Johari-Ahar M, Eghbal MA (2018). Protective effects of coenzyme Q10 nanoparticles on dichlorvos-induced hepatotoxicity and mitochondrial/lysosomal injury. Environ. Toxicol.

[17] Eghbal MA, Abdoli N, Azarmi Y (2014). Efficiency of hepatocyte pretreatment with coenzyme Q10 against statin toxicity. Arh. Hig. Rada Toksikol.

[18] Eghbal MA, Tafazoli S, Pennefather P, O›Brien PJ (2004). Peroxidase catalysed formation of cytotoxic prooxidant phenothiazine free radicals at physiological pH. Chem. Biol. Interact.

[19] Jamshidzadeh A, Niknahad H, Kashafi H (2007). Cytotoxicity of chloroquine in isolated rat hepatocytes. J. Appl. Toxicol.

[20] Eftekhari A, Azarmi Y, Parvizpur A, Eghbal MA (2016). Involvement of oxidative stress and mitochondrial/lysosomal cross-talk in olanzapine cytotoxicity in freshly isolated rat hepatocytes. Xenobiotica.

[21] Khan S, O›Brien PJ (1991). 1-bromoalkanes as new potent nontoxic glutathione depletors in isolated rat hepatocytes. Biochem. Biophys. Res. Commun.

[22] Heidari R, Babaei H, Eghbal MA (2013). Cytoprotective effects of taurine against toxicity induced by isoniazid and hydrazine in isolated rat hepatocytes. Arh. Hig. Rada Toksikol.

[23] Eskandari MR, Moghaddam F, Shahraki J, Pourahmad J (2015). A comparison of cardiomyocyte cytotoxic mechanisms for 5-fluorouracil and its pro-drug capecitabine. Xenobiotica.

[24] Reed DJ, Babson JR, Beatty PW, Brodie AE, Ellis WW, Potter DW (1980). High-performance liquid chromatography analysis of nanomole levels of glutathione, glutathione disulfide, and related thiols and disulfides. Anal. Biochem.

[25] Pourahmad J, Eskandari MR, Kaghazi A, Shaki F, Shahraki J, Fard JK (2012). A new approach on valproic acid induced hepatotoxicity: involvement of lysosomal membrane leakiness and cellular proteolysis. Toxicol. In-vitro.

[26] Marwick KF, Taylor M, Walker SW (2012). Antipsychotics and abnormal liver function tests: systematic review. Clin. Neuropharmacol.

[27] de Faria PA, Bettanin F, Cunha RL, Paredes-Gamero EJ, Homem-de-Mello P, Nantes IL, Rodrigues T (2015). Cytotoxicity of phenothiazine derivatives associated with mitochondrial dysfunction: A structure-activity investigation. Toxicology.

[28] Motohashi N, Kawase M, Satoh K, Sakagami H (2006). Cytotoxic potential of phenothiazines. Curr. Drug Targets.

[29] Zhelev Z, Ohba H, Bakalova R, Hadjimitova V, Ishikawa M, Shinohara Y, Baba Y (2004). Phenothiazines suppress proliferation and induce apoptosis in cultured leukemic cells without any influence on the viability of normal lymphocytes. Cancer Chemother. Pharmacol.

[30] Liu J, Ying M, Zhang J, Tu W, Zeng C, Wu B, Wang Q, Shen H, Zhu Z, Cai H (2018). Thioridazine upregulates programmed cell death 4 to induce apoptosis in nasopharyngeal carcinoma through the PI3K/Akt signalling pathway. Anti-Cancer Drugs.

[31] Kang S, Dong SM, Kim B-R, Park MS, Trink B, Byun H-J, Rho SB (2012). Thioridazine induces apoptosis by targeting the PI3K/Akt/mTOR pathway in cervical and endometrial cancer cells. Apoptosis.

[32] Toler SM (2004). Oxidative stress plays an important role in the pathogenesis of drug-induced retinopathy. Exp. Biol. Med.

[33] Chakraborty B, Hawes E, McKay G, Hubbard J, Korchinski E, Midha K, Choc MG, Robinson WT (1988). S-oxidation of thioridazine to psychoactive metabolites: an oral dose-proportionality study in healthy volunteers. Drug Metabol. Drug Interact.

[34] Ahmadian E, Eftekhari A, Babaei H, Nayebi AM, Eghbal MA (2017). Anti-cancer effects of citalopram on hepatocellular carcinoma cells occur via cytochrome C release and the activation of NF-kB. Anticancer Agents. Med. Chem.

[35] Dhaunsi G, Singh B, Singh A, Kirschner D, Singh I (1993). Thioridazine induces lipid peroxidation in myelin of rat brain. Neuropharmacol.

[36] Anglin R, Rosebush P, Mazurek M (2012). Psychotropic medications and mitochondrial toxicity. Nat Rev. Neurosci.

[37] Cruz TS, Faria PA, Santana DP, Ferreira JC, Oliveira V, Nascimento OR, Cerchiaro G, Curti C, Nantes IL, Rodrigues T (2010). On the mechanisms of phenothiazine-induced mitochondrial permeability transition: thiol oxidation, strict Ca 2+ dependence, and cyt c release. Biochem. Pharmacol.

[38] Laredj LN, Licitra F, Puccio HM (2014). The molecular genetics of coenzyme Q biosynthesis in health and disease. Biochimie.

[39] Jaber S, Polster BM (2015). Idebenone and neuroprotection: antioxidant, pro-oxidant, or electron carrier? J. Bioenerg. Biomembr.

[40] Kolahdouz MR, Hosseinzadeh-Attar M, Eshraghian M, Nakhjavani M, Khorami E, Esteghamati A (2013). The effect of coenzyme Q10 supplementation on metabolic status of type 2 diabetic patients. Minerva Gastroenterol. Dietol.

[41] Carvalho TC, McCook JP, Narain NR, McConville JT (2013). Development and characterization of phospholipid-stabilized submicron aqueous dispersions of coenzyme Q10 presenting continuous vibrating-mesh nebulization performance. J. Liposome Res.

[42] Kerr DS (2010). Treatment of mitochondrial electron transport chain disorders: a review of clinical trials over the past decade Mol. Genet Metab.

[43] Fard J, Hamzeiy H, Sattari M, Eftekhari A, Ahmadian E, Eghbal MA (2016). Triazole rizatriptan induces liver toxicity through lysosomal/mitochondrial dysfunction. Drug Res.

[44] Ali SA, Faddah L, Abdel-Baky A, Bayoumi A (2010). Protective effect of L-carnitine and coenzyme Q10 on CCl4-induced liver injury in rats. Sci. Pharm.

[45] Cheshchevik V, Lapshina E, Dremza I, Zabrodskaya S, Reiter R, Prokopchik N, Zavodnik IB (2012). Rat liver mitochondrial damage under acute or chronic carbon tetrachloride-induced intoxication: protection by melatonin and cranberry flavonoids. Toxicol. Appl. Pharmacol.

[46] Česen MH, Pegan K, Špes A, Turk B (2012). Lysosomal pathways to cell death and their therapeutic applications. Exp. Cell Res.

[47] Zong D, Zielinska-Chomej K, Juntti T, Mörk B, Lewensohn R, Hååg P, Viktorsson K (2014). Harnessing the lysosome-dependent antitumor activity of phenothiazines in human small cell lung cancer. Cell Death Dis.

